# TALEN-Based Gene Disruption in the Dengue Vector *Aedes aegypti*

**DOI:** 10.1371/journal.pone.0060082

**Published:** 2013-03-21

**Authors:** Azadeh Aryan, Michelle A. E. Anderson, Kevin M. Myles, Zach N. Adelman

**Affiliations:** Fralin Life Science Institute and Department of Entomology, Virginia Tech, Blacksburg, Virginia, United States of America; New Mexico State University, United States of America

## Abstract

In addition to its role as the primary vector for dengue viruses, *Aedes aegypti* has a long history as a genetic model organism for other bloodfeeding mosquitoes, due to its ease of colonization, maintenance and reproductive productivity. Though its genome has been sequenced, functional characterization of many *Ae. aegypti* genes, pathways and behaviors has been slow. TALE nucleases (TALENs) have been used with great success in a number of organisms to generate site-specific DNA lesions. We evaluated the ability of a TALEN pair to target the *Ae. aegypti kmo* gene, whose protein product is essential in the production of eye pigmentation. Following injection into pre-blastoderm embryos, 20–40% of fertile survivors produced *kmo* alleles that failed to complement an existing *kh^w^* mutation. Most of these individuals produced more than 20% white-eyed progeny, with some producing up to 75%. Mutant alleles were associated with lesions of 1–7 bp specifically at the selected target site. White-eyed individuals could also be recovered following a blind intercross of G_1_ progeny, yielding several new white-eyed strains in the genetic background of the sequenced Liverpool strain. We conclude that TALENs are highly active in the *Ae. aegypti* germline, and have the potential to transform how reverse genetic experiments are performed in this important disease vector.

## Introduction

Vector-borne diseases such as malaria and dengue fever remain large public health burdens, and novel interventions are still needed. The development of new methods of vector control would be aided substantially by a more detailed genetic and biochemical understanding of many critical behaviors such as development, host seeking, bloodfeeding and vector competence. Though the genomes of several disease vector mosquitoes have been sequenced, many mosquito-specific genes remain without any functional annotation, and there is much still to be learned with regards to understanding the genetic basis for these key behaviors. Of the disease vector mosquitoes that have a sequenced genome, *Aedes aegypti*, the primary vector for dengue viruses, is probably the most tractable due to the ease of adapting new strains to the laboratory environment and the ability to delay the hatching of developed embryos for months at a time. Progress in the field of site-specific gene editing with meganucleases indicates that these tools are sufficiently mature as to provide a novel means of performing reverse genetic experiments in a range of non-traditional organisms, including *Ae. aegypti*.

Though other meganucleases such as homing endonucleases and zinc finger nucleases have been used to perform custom editing of various genomes (reviewed in [1], [2]), their adoption by the research community has been limited at best. Limitations with these systems relate to the difficulty of assembling/reengineering these molecules to recognize new target sites due to the strong context-dependence of their DNA-binding regions. In contrast, transcription activator-like elements (TALEs) from the plant pathogenic bacteria *Xanthomonas* contain a simple, context independent DNA binding region [3], [4]. In these molecules, DNA binding is conferred by a series of 34 amino acid repeats, differing only at two positions (the repeat variable diresidue, or RVD), where each RVD specifies a given target nucleotide [3], [4]. Fusion of TALE repeat domains to the *Fok*I nuclease domain confers extreme site specificity and has allowed the editing of a number of diverse genomes (reviewed in [1], [5]), including the insects *Drosophila melanogaster* [6], *Bombyx mori* [7], [8] and *Gryllus bimaculatus* [9]. However, at present there are no reports of TALE nuclease editing in any disease vector species.

To examine the possibility of using TALE-based nucleases to edit the *Ae. aegypti* genome, we sought to take advantage of a known physical mutant with a clearly defined and easily recognizable phenotype [6], [7], [8]. While many physical mutants for this mosquito have been described (reviewed in [10]), few have been associated with a specific gene product. A white-eyed mutant strain [11] was hypothesized to be orthologous to the Drosophila *cinnabar* (*cn)* mutant; later work confirmed that eye pigmentation in this strain could indeed be complemented by the Drosophila *cn+* gene both transiently and through stable germline transformation [12], [13], [14]. This strain, first identified as *w*, but now known as *kh^w^* [12], is used routinely in our lab as a convenient recipient for transgene insertions [15], [16] as the lack of eye pigment facilitates screening using the eye-specific 3xP3 synthetic promoter [17]. *kh^w^* strain mosquitoes are deficient in kynurenine 3-monoxygenase (KMO) activity, and thus fail to produce ommochromes from tryptophan precursors [11], [12], [18].

We found that TALEN-based targeting of the *Ae. aegypti kmo*+ allele was a highly efficient process, with 20–40% of fertile G_0_ females producing new *kmo* mutant alleles in a complementation assay with the *kh^w^* strain. Mutation rates were sufficiently robust that blind G_1_ intercrosses resulted in several new white-eyed strains (Lvp*^kmo^*) developed entirely within the genetic background of the sequenced Liverpool (Lvp) strain of *Ae. aegypti*. These results suggest that TALE-based applications are poised to revolutionize the study of *Ae. aegypti* genetics and allow the development of new genetic methods to disrupt disease transmission by this important mosquito vector.

## Results

### Selection of TALEN target site and transient embryo assay

**Figure 1:**
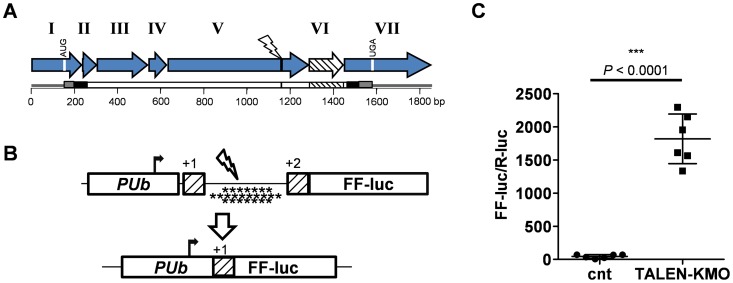
Plasmid-based SSA assay for TALEN activity in *Ae. aegypti* embryos. (**A**) cDNA structure of the *Ae. aegypti kmo* gene (AAEL008879). Exons (roman numerals), initiation and termination (white vertical bars) codons, and TALEN recognition site (black vertical bar) are indicated. The exon skipped in *kh^w^* strain is indicated (white, cross-hatched arrow). The KMO ORF, with predicted extracellular (grey), transmembrane (black) and intracellular (white) domains are indicated below. (**B**) Schematic representation of the SSA test plasmid. TALEN recognition sites for *Ae. aegypti kmo* were located between two direct repeats (cross-hatched boxes) of the initial 298 bp of the Firefly luciferase (FF-luc) coding region. Stop codons (denoted by *) in the +1 (7), +2 (10) and +3 (7) reading frames in the spacer are indicated. Transcription from the *polyubiquitin* (*PUb*) promoter is expected to lead to translation in the +1 ORF at the FF-luc AUG in the first repeat, resulting in a truncated protein. Fourteen additional AUG codons are present prior to the full-length +2 frame FF-luc ORF to minimize read-through translation. Double-stranded DNA break induction by the introduced TALEN pair (lightning shape) followed by SSA-mediated repair restores the FF-luc ORF. (**C**) Relative levels of FF-luc activity in the presence or absence of the KMO-targeted TALEN pair 24 hours following injection into *Ae. aegypti* embryos. Statistical significance following the Mann-Whitney test is indicated.

Full-length cDNAs for both the wt and *kh^w^* (*kmo*) gene (AAEL008879) have been characterized, with an in-frame deletion of 162 bp implicated as the causative mutation in the *kh^w^* strain [18]. The KMO protein is predicted to contain transmembrane domains near both the N and C termini, with the majority of the protein located on the cytoplasmic face of the membrane (Fig. 1A). Alignment of the *kmo* cDNA described by Han et al [18] to the *Ae. aegypti* genome assembly revealed a structure consisting of seven exons (Fig. 1A). Interestingly, the proposed 162 bp deletion corresponded precisely to exon 6, suggesting that the *kh^w^* phenotype may in fact be due to the failure to correctly splice in this exon. Indeed, sequencing of genomic DNA from this region from both *kh^w^* and Lvp strain mosquitoes revealed an 11 bp deletion in the splice acceptor site of exon 6 only in the *kh^w^* strain (Fig. S1). As the loss of exon 6 was sufficient to eliminate KMO activity, we designed our TALEN pair to cleave the region just upstream of the exon 5–6 junction. A frameshift mutation at this location would be expected to result in the loss of coding information present in both exons 6 and 7, including the C-terminal membrane spanning domain.

To screen our TALEN pair for activity in *Ae. aegypti* embryos, we inserted the ∼50 bp TALEN target site from the *Ae. aegypti kmo* gene into a firefly luciferase-based reporter construct containing a tandem duplication of the first ∼300 bp of the luciferase open reading frame (Fig. 1B). Successful TALEN-based cleavage at the target site, followed by single-strand annealing (SSA) repair is expected to result in the collapse of the two direct repeats and thus translation of the full length luciferase protein (reviewed in [19]). Indeed, following injection into pre-blastoderm embryos, we observed strong activation of firefly luciferase activity (Fig. 1C). We conclude that TALE-based nucleases are active in the early embryo of *Ae. aegypti* mosquitoes.

### Identification of new TALEN-generated kmo alleles through lack of complementation with kh^w^

To detect heritable gene editing, we injected the *kmo*-targeting TALEN pair into pre-blastoderm embryos of the black-eyed Liverpool (Lvp, *kmo*+/*kmo*+) strain and screened the progeny of the surviving individuals for white eyes. As the *kh^w^* phenotype is completely recessive, injected survivors were mated to *kh^w^* (*kmo^w^*/*kmo^w^*) mosquitoes in order to detect new mutant alleles. A test cross between untreated Lvp and *kh^w^* strains demonstrated that 100% of progeny retained wild-type eye color (Table 1), confirming that our Lvp strain was free from rare *kmo* mutant alleles that might otherwise go undetected. In contrast, following injection of the TALEN constructs, white-eyed progeny were identified in seven of nine pools in experiment 1, and all three pools in experiment 2 (Table 1).

**Table 1:** Generation of new mutant *kmo* alleles from pooled G_0_ populations.

Exp.	# embryos injected	# G_0_ (%)	G_0_ gender	Pool ID	Phenotype*		
					wt	we	%we
Neg. control	n.a.	n.a.	n.a.	n.a.	8970	0	0
#1	1020	187 (18.3%)	♀	P1	1200	64	5.3%
			♀	P2	1350	70	5.2%
			♀	P3	250	24	9.6%
			♂	P4	1700	56	3.3%
			♂	P5	900	23	2.6%
			♂	P6	1100	11	1.0%
			♂	P7	2400	0	0%
			♂	P8	2700	0	0%
			♂	P9	1400	56	4.0%
#2	1010	195 (19.3%)	♀	B1	1800	130	7.2%
			♀	B2	200	31	15.5%
			♀	B3	36	3	8.3%

* wt, wild-type; we, white-eyed.

**Figure 2:**
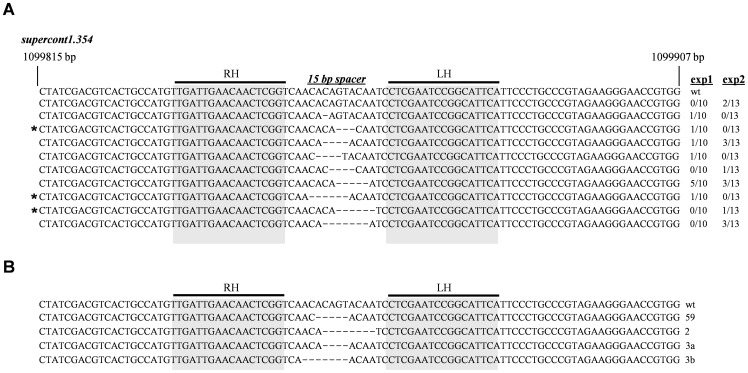
TALEN-induced deletions in AAEL008879, the *Ae. aegypti kmo* gene. Sequenced amplicons obtained from white-eyed individuals were aligned and compared to the wt *kmo* sequence in the Lvp/*kh^w^* hybrid genetic background (**A**) or the Lvp background alone (**B**). The DNA-binding regions of the right (RH) and left (LH) TALENs are indicated. The three in-frame deletions are indicated (*).

Since most of the pools produced white-eyed progeny, it seemed likely that by pooling G_0_ individuals (a strategy common in *Ae. aegypti* transgenic experiments, due to the low rate of transposon-based transformation) we may have been underestimating the rate of TALEN-based editing. All six female pools were given a second bloodmeal, after which fed female mosquitoes were transferred to single rearing tubes and allowed to deposit eggs individually. From 65 fertile G_0_ females, we obtained 23 that produced white-eyed progeny, an editing rate of ∼35% (Table 2). This is an order of magnitude greater than transposable-element transformation in this species and confirms that our initial pooling strategy underestimated the amount of editing by a factor of four. Individual females produced an average of 38% white-eyed progeny, with some females producing up to 75% (Table 3). Sequencing of the *kmo* target site from each of these families confirmed the existence of deleted bases (1–7 bp) in 21 of 23 cases (91%) (Fig. 2A). The remaining two cases may represent larger deletions that spanned at least one of the PCR primers, allowing amplification of only the *kh^w^* allele. While most (18/21, 86%) of the deletions recovered represented frame-shift mutations, three in-frame deletions were also found: ΔThr^337^, ΔThrVal^337–8^, and ΔCysThr^336–7^, suggesting a potential critical role for these residues in KMO activity or stability. Based on these data, we conclude that TALEN-based gene editing is a highly efficient process in *Ae. aegypti*.

**Table 2:** Frequency of TALEN-generated *kmo* alleles per fertile G_0_ female.

	G_0_ ♀ (total)	G_0_ ♀ (fertile)	# G_0_ ♀ producing *kmo* progeny	TALEN frequency
P1	30	12 (40%)	3	25%
P2	30	18 (60%)	6	33%
P3	25	6 (24%)	1	17%
Exp1 total	85	36 (42%)	10	28%
B1	35	12 (34%)	6	50%
B2	30	11 (37%)	5	45%
B3	27	6 (22%)	2	33%
Exp2 total	92	29 (32%)	13	45%

**Table 3:** Generation of new mutant *kmo* alleles from single G_0_ females*.

G_0_ ♀**	P1			P2			P3			B1			B2			B3		
	wt	we	%	wt	we	%	wt	we	%	wt	we	%	wt	we	%	wt	we	%
1	7	14	67%	15	28	39%	52	3	6%	43	28	39%	9	13	59%	36	18	33%
2	16	3	17%	17	5	13%				35	5	13%	46	1	2%	20	8	29%
3	8	24	75%	39	7	20%				29	7	20%	6	5	46%			
4				46	27	59%				19	27	59%	18	6	25%			
5				34	9	23%				30	9	23%	36	8	18%			
6				27	9	41%				13	9	41%						

* wt, wild-type; we, white-eyed.

** Each row represents the 1^st^, 2^nd^, 3^rd^, etc... female in each pool that produced one or more *kmo* mutant progeny.

### Identification of new kmo alleles in a complete Lvp genetic background

**Figure 3:**
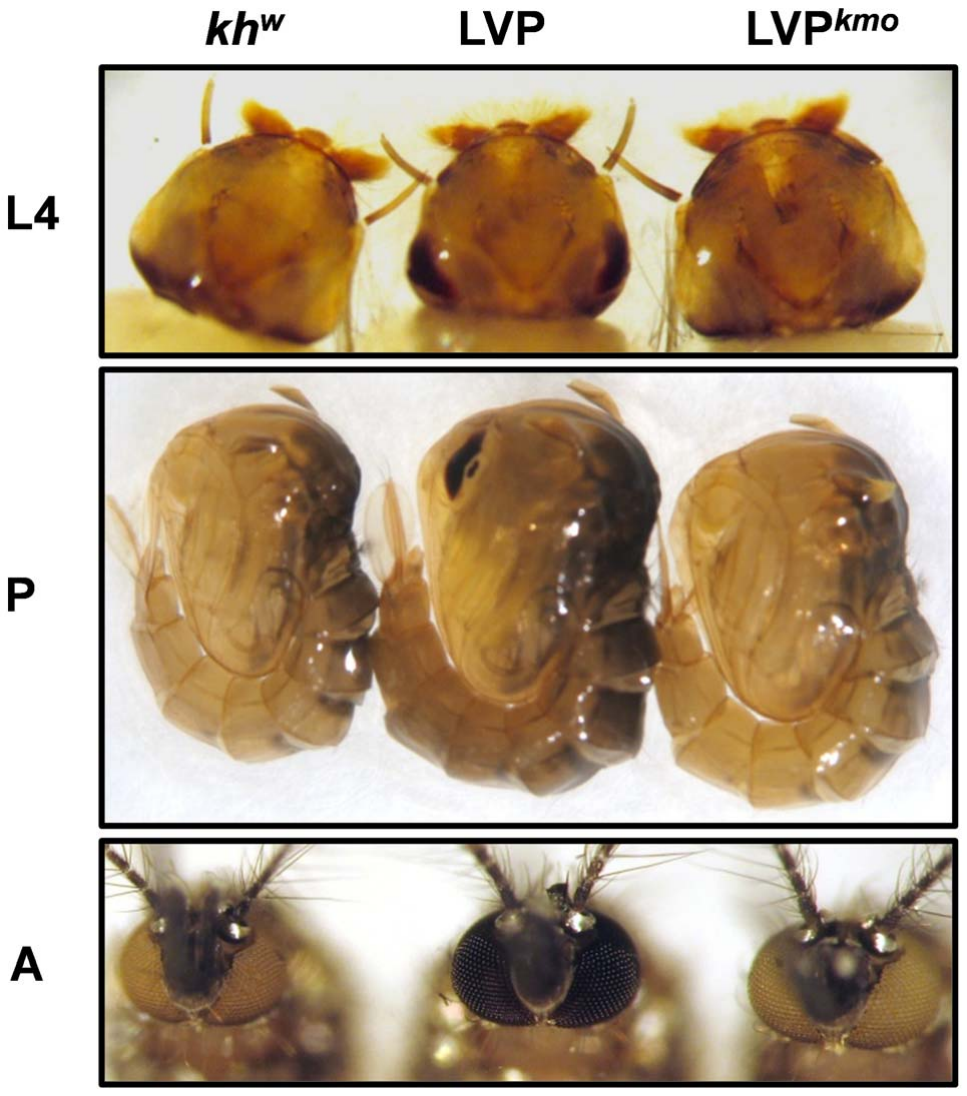
TALEN-generated *kmo* alleles phenocopy *kh^w^* strain mosquitoes. LVP, *kh^w^* and LVP*^kmo^* mosquitoes imaged as larvae (L4), pupae (P) and adults (A).

The identification of new *kmo* mutant alleles in the above experiments was simplified through the use of an existing mutant strain that failed to provide complementation. However, such a luxury would not be found in most circumstances, where investigations will focus on targeting new genes in order to identify novel phenotypes. Likewise, gene editing experiments will likely need to be performed entirely within the strain of study, without the introgression of confounding genetic material from unrelated and highly inbred strains. To determine if we could identify novel *kmo* mutations without the assistance of the *kh^w^* complementation assay, we injected the *kmo*-targeting TALEN pair into Lvp embryos, and this time backcrossed the surviving individuals to Lvp strain mosquitoes. Offspring from this cross were 100% black-eyed; siblings within each family were intercrossed to obtain G_2_ progeny. From just 10 fertile G_0_ founders, we identified three that produced white-eyed progeny in the G_2_ generation (Table 4). The frequency of white-eyed individuals in the G_2_ generation ranged from 4.6–10.4%. This is consistent with an initial mutant allele frequency of 21–32% in the G_1_ generation, similar to our prior experiments (Table 3). Sequencing of the TALEN target site in white-eyed G_2_ individuals revealed genetic lesions consistent with a loss of function phenotype in all cases (Fig. 2B). In fact, we recovered four independent lesions from these three founders, suggesting that a single individual male produced multiple sperm with independent deletion events. Phenotypically, *Lvp^kmo^* individuals were indistinguishable from *kh^w^* strain mosquitoes at all life stages (Fig. 3). Thus, we conclude that TALENs can be used to edit the *Ae. aegypti* genome in a strain-independent manner at high efficiency, and that individuals homozygous for an expected mutation can be recovered at the G_2_ stage at useful frequencies, even in the absence of any screening at the G_1_ (hemizygous) state.

**Table 4:** Identification of new *kmo* mutant alleles in the LVP genetic background.

ID#	G_0_ gender	G_2_ eye phenotype		
		wt	we	%we
1	♀	1700	0	
2	♀	1500	105	6.5%
3	♂	950	110	10.4%
5	♂	650	0	
9	♂	1050	0	
12	♂	750	0	
16	♂	450	0	
36	♂	650	0	
50	♂	800	0	
59	♂	750	36	4.6%

## Discussion

Research efforts using model organisms such as *D. melanogaster*, *S. cerevisiae*, *C. elegans* and *A. thaliana* have benefitted tremendously from the availability of genetic stock centers housing large collections of mutant strains; whereas reverse genetic experiments in non-model organisms have been more limited. While the development of RNAi technology has enabled some such experiments to move forward, this technology is limited by low penetrance of injected double-stranded RNA into some tissues [20], gene by gene variation in the degree and timing of knockdown (Adelman, unpublished observations), and off-target effects resulting from the large pool of siRNAs generated from the introduced precursor molecules [21]. In contrast, the ability to directly and specifically disrupt a gene of interest offers the possibility to perform intricate reverse genetic experiments on any gene, in any organism. We confirm that TALEN-based gene disruption can be a highly efficient process in *Ae. aegypti*, with editing rates between 20–40%. This is an order of magnitude greater than both traditional transposon-based transformation [22] and phiC31-based recombination [23], and offers up the possibility that TALE-based experiments will be much more amenable to moderate or higher throughput applications than what has been achieved over the past decade with these less robust genetic systems.

In our experiments, we only examined a single TALEN pair. Thus, it is possible that not every such pair will achieve the same or similar activity. However, the success rates we observed are similar to those described in many other organisms, including several other insects [6], [8], [9]. Given the success of others in large scale TALEN pairs screens [24], [25], [26], [27], [28], the rate of TALEN failure appears to be acceptably low (<20%). The primary difficulty with developing new TALEN pairs to target genes of interest is the time and effort required to assemble the numerous TAL repeat constructs. However, the recent availability of many new assembly methods have substantially decreased the time required for developing new TALEN pairs, with full assembly decreasing from 6–8 weeks to 3–24 hrs [24], [27], [29], [30]. We anticipate that if need be groups of TALEN pairs can be screened initially using the SSA assay we described in pre-blastoderm embryos. Others have demonstrated that in vitro SSA results are highly correlated with germline editing activity [7], [31]; we have made similar observations with homing endonucleases in *Ae. aegypti* (Adelman, unpublished). Thus, germline editing experiments can be restricted to those TALEN pairs which perform well in this assay.

TALEN-based editing was associated with small deletions ranging in size from 1–7 bp. This is similar to results obtained in other insects such as the vinegar fly *D. melanogaster* [6], the silkworm *B. mori* [7], [8] and the cricket *G. bimaculatus* [9]. This limited deletion size has several favorable consequences; the most significant in our opinion is that we were able to recover essentially the same set of deletions in two independent experiments, where identical 4-bp and 5-bp deletions were recovered in both instances. This indicates that there may be no substantial burden for the long-term maintenance of TALEN-modified mosquito strains. Thus, there is no need for large (expensive) stock centers to house an ever-growing collection of TALEN-modified strains. As long as the TALE-binding sites are made available (or the TALEN constructs themselves), the disruption in question could be re-generated at any point in the future, in the most useful genetic background at the time. In the same vein, identical deletions obtained from separate founders could be mixed into a single population, substantially eliminating the influence of any off-target effects possibly occurring within a single founder.

The modularity of TALE-binding domains lends them to applications beyond the generation of double-stranded DNA breaks. Though not addressed directly in our experiments, our data indicate that TALE fusions to other active domains, such as transcriptional activators/repressors [32] or recombinases [33], are certainly worth pursuing in *Ae. aegypti*. Likewise, experiments involving the knock-in of a transgene [31] or single-stranded oligonucleotide [29], [34] through homologous recombination may further increase the ever growing utility of TALE-based enzymes in specifically editing the genome of this mosquito.

## Materials and Methods

### Plasmid construction

To generate the SSA reporter, a synthetic fragment encoding the first 298 bp of the Firefly luciferase gene and an additional 354 bp spacer region was inserted in between the *PUb* promoter and FF-luc ORF of pGL3Basic/*PUb*-FFluc [16]. The spacer region included a portion of the *Ae. aegypti kmo* gene containing the target site. TALEN constructs were obtained from Cellectis Bioresearch (Paris, France). Each TALEN-encoding sequence was placed downstream of the *Ae. aegypti polyubiquitin* promoter through standard cloning procedures. DNA for each of the *PUb*-TALEN plasmids was prepared using the Qiagen Endo-free Maxi-prep kit (Experiment #1) or the Machery-Nagel endo-free midi kit (Experiment #2) as directed by the manufacturer prior to injection into mosquito embryos.

### Mosquito rearing, crosses, and embryonic injections

*Ae. aegypti* mosquitoes (Lvp and *kh^w^* strains) were maintained in an insectary at 28°C and 60–70% humidity, with a 14/10 h day/night light cycle. Embryonic injections were performed as described previously [15]. For the transient assay, an injection mix containing the SSA test construct, *PUb*-TALEN and a normalization control in injection buffer [14] were introduced into ∼1 hr old pre-blastoderm embryos. All plasmids were present at 0.2 µg/µl, for a total DNA concentration of 0.8 µg/µl. Embryos were snap-frozen in liquid nitrogen at 24 hours post injection and lysate prepared for dual luciferase assay (Promega, Madison, WI). Luciferase activity was determined using the Dual-Luciferase Reporter Assay System with a GloMax-Multi Detection System instrument according to the manufacturer's instructions (Promega, Madison, WI). For germline experiments, *PUb*-TALEN constructs (0.3 µg/µl of each) were similarly introduced into developing embryos. G_0_ survivorship counts were based on the number of individuals emerging as adults. For mating, G_0_ survivors were separated into single vials as pupae; emergent adults were collected each day and transferred into male-only or female-only cages. G_0_ males were anesthetized under CO_2_ and mated individually to 5 virgin *kh^w^* or Lvp strain females for two to three days, at which point they were either directly offered a bloodmeal (for Lvp experiments) or combined into families. Groups of G_0_ females were combined with 15–20 males of the appropriate parental strain prior to bloodfeeding and egg collection.

### PCR and mutational analysis

Primers 5′-TCAACATAATTATACATGGCCAGATCGCAG-3′ and 5′-TCTGATTGGTCGTGAGCGGTTGGTTAAGGA-3′ were used to amplify the region containing the *kmo* target site from wild-type individuals or from TALEN-injected progeny. PCR was performed using the Phire Animal Tissue Direct PCR kit (Thermo Scientific, Lafayette, CO) using either a portion of the larval body in dilution buffer or an adult leg placed directly in the master mix as described by the manufacturer. Amplification conditions were: 98°C for 5 min, 98°C for 5 s, 70°C for 5 s, 72°C for 20 s, 39 cycles, 72°C for 1 min. Where amplification was unsuccessful, a second set of primers was used under the same conditions (5′-TCCAACGACGAAGGAATCTACTC-3′ and 5′-CAAAACGACCGCATACAAAAC-3′). All amplicons were purified and sequenced directly in both directions using the same primers used during the PCR step.

## Supporting Information

Figure S1 **The** ***kh^w^*** **phenotype is due to exon skipping.** Sequences obtained following PCR of the intron 5-6/exon 6 genomic interval of gene AAEL008879. Coordinates on supercontig1.354 are given. The splice acceptor site is highlighted in yellow; the final AG of the intron is indicated in bold.
(TIF)
