# Supplementary figures and images for: TALEN-Based Gene Disruption in the Dengue Vector Aedes aegypti
Source: PLoS One. 2013 Mar 21;8(3):e60082. doi: 10.1371/journal.pone.0060082 (PMC3605403; doi:10.1371/journal.pone.0060082)

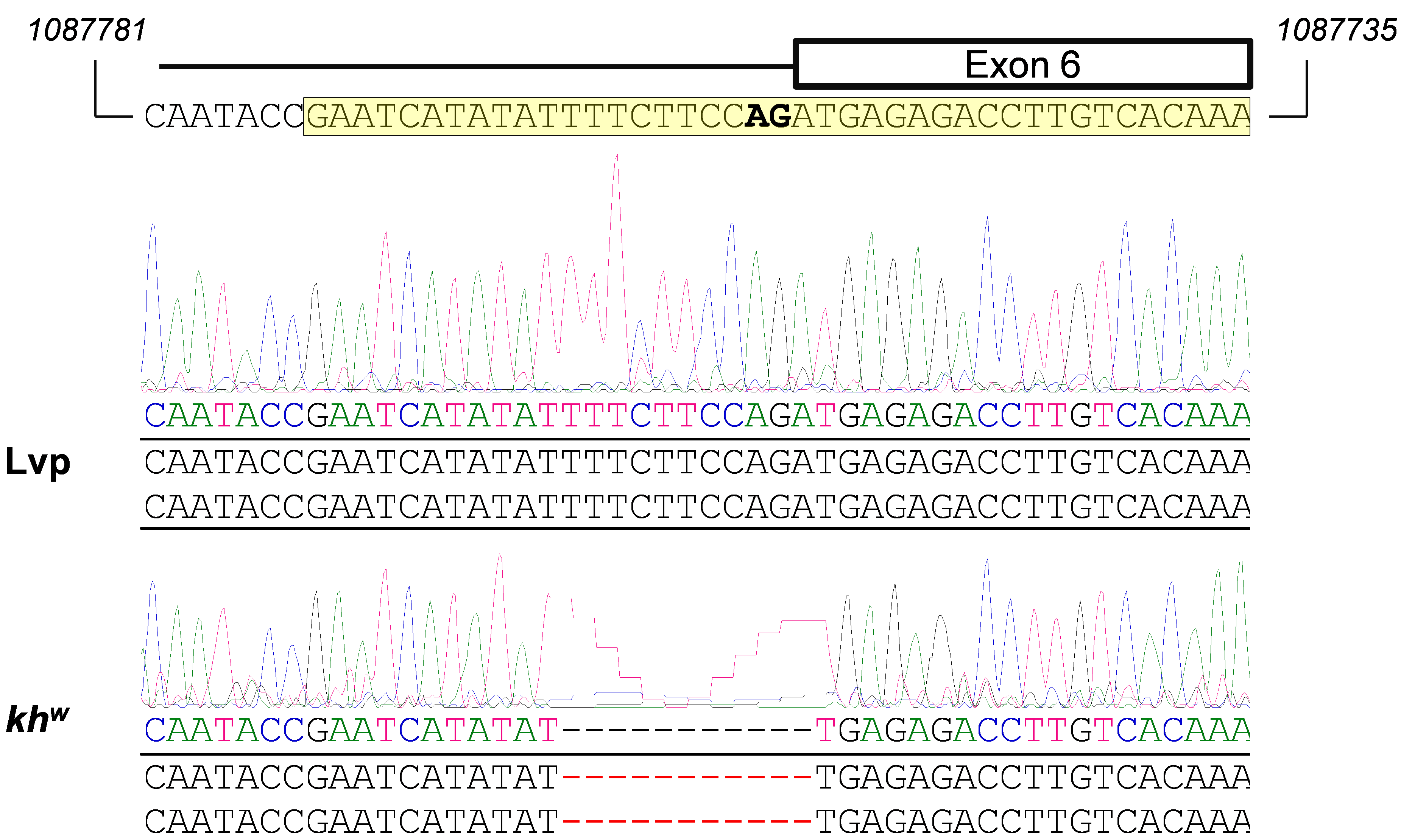

Supplement: Figure S1 — The khw phenotype is due to exon skipping. Sequences obtained following PCR of the intron 5-6/exon 6 genomic interval of gene AAEL008879. Coordinates on supercontig1.354 are given. The splice acceptor site is highlighted in yellow; the final AG of the intron is indicated in bold. (TIF) [file pone.0060082.s001.tif]
